# Small fiber neuropathy with dysautonomia and positive GPCR and FGFR-3 antibodies after the first BNT162b2 dose

**DOI:** 10.1016/j.clinsp.2024.100557

**Published:** 2025-01-27

**Authors:** Josef Finsterer, Carla Alexandra Scorza, Fulvio Alexandre Scorza, Ana C. Fiorini

**Affiliations:** a Neurology Neurophysiology Center, Vienna, Austria; b Escola Paulista de Medicina, Universidade Federal de São Paulo (EPM/UNIFESP), São Paulo, SP, Brazil; c Programa de Estudos Pós-Graduado em Fonoaudiologia, Pontifícia Universidade Católica de São Paulo (PUC-SP), Departamento de Fonoaudiologia, Escola Paulista de Medicina/Universidade Federal de São Paulo (EPM/UNIFESP), São Paulo, SP, Brazil

Small Fiber Neuropathy (SFN) is a relatively common complication of SARS-CoV-2 Infection (SC2I), but also of SARS-CoV-2 Vaccination (SC2V).^1^^,^^2^ In general, SFN is due to the involvement of the A-delta and C fibers and manifests clinically as sensory disturbances, pain, or autonomic dysfunction in a highly variable distribution.^3^ The large fibers are usually not affected, but both large fibers and SFN can coexist.^3^ SFN can be primary (genetic) or secondary to various metabolic, infectious, immunologic, toxic, or paraneoplastic causes. SFN secondary to SC2V has been repeatedly reported,^4^ and there are no differences compared to SC2I-related SFN in clinical presentation, course, or treatment effect. In some cases, SC2I-associated SFN is related to autoantibodies against various human targets.^5^ One patient with SFN associated with antibodies against G Protein-Coupled Receptors (GPCR) and FGF Receptor-3 (FGFR3) after the first dose of BNT162b2 has not yet been reported.

The patient is a 24-year-old woman who developed postural tachycardia syndrome (POTS) due to SFN four weeks after administration of the first BNT162b2 dose in August 2021. She also tested positive for antibodies against GPCR and PGFR3 (Table 1). Just one day after vaccination, she had developed fever, pain, tingling, hypoesthesia, dysesthesias, and burning paresthesias in the upper and lower limbs with left-sided dominance, although the hands and feet were initially spared. Two days after the vaccination, she also noticed paresthesias in the spine. The flu-like symptoms persisted for 1 week. Four weeks after the vaccination, the paresthesias also affected the face, hands, and feet (burning). Glucocorticoids were ineffective. Nine months after vaccination, the patient developed sinus tachycardia of up to 160 bpm. The cardiologic examination revealed a POTS. Pre-vaccination history revealed only lactose intolerance, which was diagnosed at the age of 22. The only medication she took regularly before the vaccination was the pill.

**Table 1:** GPCR and FGFR3 autoantibodies in the index patient.

Antibodies	Reference limit	11.8.22
Anti-AT1R	> 17 U/mL	13.2
Anti-ETAR	> 17 U/mL	14.3
Anti-β1 adrenergic	> 15 U/mL	28.7
Anti-β2 adrenergic	> 14 U/mL	29.7
Anti-MCR2	> 9.0 U/mL	10.2
Anti-FGFR3	> 12 U/mL	36.9
Anti-TSHDS IgM	> 9 U/mL	4.0

MCR2, Anti-muscarinic Cholinergic Receptor-2.

Clinical neurological examination was inconclusive, but quantitative sensory testing revealed marked dysfunction of the A-delta and C fibers and mild dysfunction of the A-beta fibers. Magnetic Resonance Imaging (MRI) of the brain (3 times) showed only a non-specific lesion in the tectum. MRI of the cervical spine (once) and the entire spine (once) was inconclusive. Repeated Nerve Conduction Studies (NCS) ruled out large fiber neuropathy. Visual Evoked Potentials (VEPs), Magnetic Evoked Potentials (MEPs) and Cerebrospinal Fluid (CSF) examinations were inconclusive. The Sudoscan showed significantly reduced sweat production of the plantar eccrine sweat glands. Tibial Somato-Sensory Evoked Potentials (SSEP) revealed a normal N40 latency twice on both sides, but a difference between the left (24.5 ms) and right (23.2 ms) sides. A skin biopsy from the thigh and distal lower limb revealed a normal Intraepidermal Nerve Fiber Density (IENFD) of 8.9 fibers/mm^2^) in the thigh, but a reduced IENFD of 6.2 fibers/mm^2^ in the distal lower limb.

Total IgG, Interleukin-6 (IL-6), Brain Natriuretic Peptide (proBNP), Troponin-T (TpT), cholesterol, HDL, LDL, triglycerides, liver function parameters (AST, ALT, bilirubin, gammaGT) were all normal. The antinuclear antibodies were increased to 1:320. GPCR-IgG antibodies against the Angiotensin-1 Receptor (AT1R) and the Endothelin-A Receptor (ETAR) were negative (Table 1). GPCR-IgG antibodies against Adrenergic Alpha-1 and Alpha-2 Receptors (A1ADR, A2ADR), Adrenergic Beta-1 and Beta-2 Receptors (B1ADR, B2ADR), muscarinic acetylcholine receptors type 1–5 (M1R-M5R), Angiotensin-Converting Enzyme-2 (ACE2) and the MAS proto-oncogene (MASR1) were positive (Table 1). The determination of IgM TSHDS and FGFR3 antibodies was also positive (Table 1). Cardiologic evaluation by clinical examination, echocardiography (2-times), 24 h ECG, Schallong test and tilt table test revealed POTS. The patient was treated with ivabradine (5 mg/d) and an ointment containing ketamine and amitriptyline.

The index patient is of interest because SFN is associated with antibodies against GPCR and FGFR3 after the first dose of the BNT162b2 vaccine. A causal relationship is supported by the fact that SFN has been repeatedly reported after SC2V and is an established complication of SC2V,^1^ that SFN developed temporally coupled to the vaccination, that other patients with SFN and positive GPCR antibodies have been reported^6^ and that all other possible causes of secondary SFN have been thoroughly excluded. The patient is also interesting because the SFN occurred not only with sensory disturbances but also with POTS. POTS is a common manifestation of SFN but has also been reported once due to SC2V.^4^ SFN and POTS have been reported not only after SC2V but also after vaccinations against human papillomavirus, varicella-zoster virus, Lyme and rabies.^7^

In general, neuropathy following vaccination is likely to be immune-mediated and is due to either hypersensitivity to the solvent of the vaccine or to the active components of the vaccine.^6^ The presence of ACE2 antibodies in the index patient indicates an immune response to the active vaccine, as the vaccine mRNA codes for the spike protein that binds to the ACE2 receptors. Sometimes autoantibodies result from a general activation of the immune system and are not specific to the disease. In the index patient, however, the presence of GPCR antibodies suggests that there was an immune-mediated component to the disease, but it remains speculative whether these autoantibodies directly caused the symptoms. One argument that GPCR antibodies may have caused the symptoms is that they directly target receptors normally involved in the blood pressure and heart rate responses that are impaired in POTS. Antibodies against FGFR-3 have been detected in 28 % of patients with SFN and dysautonomia.^8^ GPCR antibodies have also been associated with chronic fatigue, fibromyalgia and post- and long-COVID syndrome.^9^

Several approaches have been proposed for the treatment of SC2V-associated SFN. In a pilot study of 8 patients with TS-HDS-associated or FGFR3-associated SFN, the administration of IVIG provided no significant benefit for the cohort studied.^10^ Another patient with GPCR antibody-associated SFN also received IVIG, but developed hemolytic anemia.^6^ However, the patient reported by Schelke et al. benefited significantly from plasma exchange.^6^ In this patient, the GPCR antibody titers decreased significantly and the patient's tinnitus also partially disappeared.^6^ In contrast to the index patient, the patient reported by Schelke et al. developed SFN and POTS only after the second BNT162b2.^6^

In summary, this case shows that SFN with antibodies against GPCR and FGFR3 can be a complication of SC2V, clinically manifesting with sensory disturbances, pain, and POTS. Symptomatic treatment of SFN can be very effective, but immunomodulatory treatment may be of limited effect.

## Ethical approval

Has been provided by the institutional review board.

## Consent to participation

Not applicable.

## Consent for publication

The patient consented to the publication.

## Availability of data and material

All data are available from the corresponding author.

## Authors’ contributions

CS, FS, AF: formal analysis, valudation, writing- review and editing.

JF: conceptualisation, data curation, formal analysis, valudation, writibg-oroiginal draft, writing- review and editing.

## Declaration of competing interest

The authors declare that the research was conducted in the absence of any commercial or financial relationships that could be construed as a potential conflict of interest.
